# Agrestal Environments and Maternal Genetic Effects Weaken the Ecological Barriers for Crop to Wild Introgression in Sunflower

**DOI:** 10.1002/ece3.72961

**Published:** 2026-02-03

**Authors:** Ignacio J. Fanna, Fernando Hernández, Kristin L. Mercer, Alejandro Presotto

**Affiliations:** ^1^ Departamento de Agronomía Universidad Nacional del Sur Bahía Blanca Argentina; ^2^ Centro de Recursos Naturales Renovables de la Zona Semiárida Consejo Nacional de Investigaciones Científicas y Técnicas Bahía Blanca Argentina; ^3^ Department of Botany and Biodiversity Research Centre University of British Columbia Vancouver British Columbia Canada; ^4^ Department of Horticulture and Crop Science The Ohio State University Columbus Ohio USA; ^5^ Institute of Sustainable Agroecosystem Services University of Tokyo Tokyo Japan

**Keywords:** agrestal, crop‐wild hybridization, fitness, *Helianthus annuus*, maternal effect, ruderal

## Abstract

Admixture between crops and their wild relatives may lead to the evolution of aggressive weeds in agricultural and natural habitat. As many crops and their wild relatives belong to the same species, admixture is common, and the low fitness of crop‐wild hybrids is the main ecological barrier for introgression. However, fitness of hybrids can vary between environmental conditions, genetic background of recipient and donor populations, and maternal genetic effects. Here, we study how these three factors influence the phenotypic variation of crop‐wild sunflower hybrids in the field. To this end, we measured seven variables related to fitness throughout the life cycle in eight populations (including reciprocal crop‐wild hybrids and their parents) in two agrestal (wheat and maize plantings) and one ruderal (natural vegetation in a human‐disturbed area) environments over 2 years. Overall, high out‐of‐season germination was observed in the first year but declined in the second across all environments. In ruderal habitats, flowering was delayed, and fitness was lower compared to agrestal ones. Hybrids exhibited similar or lower fitness than wild plants in agrestal habitats, but the differences were larger in ruderal habitats. Hybrids with maternal crop parents displayed higher fitness than their reciprocal crosses across all environments in the first year, but differences disappeared the second year. Our findings suggest that agrestal conditions and crop maternal parent favor the establishment of crop‐wild hybrids, weakening the ecological barriers for crop introgression.

## Graphical abstract

We highlight how agrestal environments and maternal effects may facilitate the adaptation of crop‐wild hybrid sunflower to agroecosystems. We present two consecutive years of data on diverse wild and reciprocal crop‐wild hybrid sunflower populations planted in three contrasting, ruderal or agrestal, environments. We found that (i) agrestal environments are more favorable for crop introgression than ruderal environments, (ii) plant dynamics throughout the life cycle differ between ruderal and agrestal environments, and (iii) crop‐wild hybrids may evolve differently due to maternal effects under specific competitive conditions.

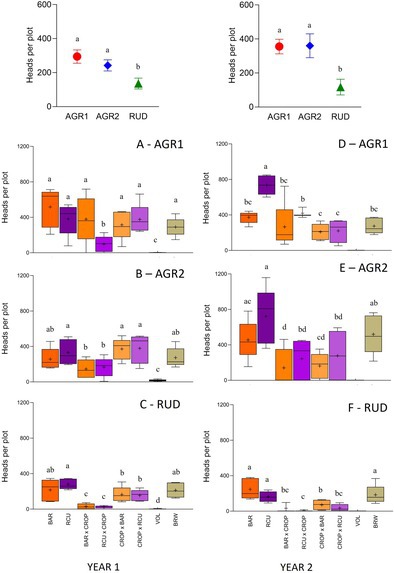

## 1 Introduction

Pollen‐mediated gene flow is one of the main forces driving evolution (Ellstrand 2003), resulting when organisms of different taxa reproduce to form progeny with genetic information from both parents (Mason 2016). Unlike mutations, which involve genetic changes in one (or a few) nucleotide at a time, admixture modifies an array of alleles simultaneously. If admixture proceeds, some alleles may eventually introgress (i.e., be permanently incorporated into one genome from the other) as has occurred in several crops (Ellstrand et al. 2013; Suarez‐Gonzalez et al. 2018; Vercellino, Hernández, Pandolfo, et al. 2023). Introgressed genetic variants may enable populations to overcome adaptive boundaries, promoting range expansion, local adaptation, colonization of new habitats, or even speciation (Abbott et al. 2013; Harrison and Larson 2014; Seehausen 2004). Admixture also may increase genetic variation of recipient populations, create novel genotypes, and increase invasiveness of introduced species (Li et al. 2018; Shi et al. 2018; Hernández et al. 2024).

Permanent introgression of cultivar alleles into wild relatives has been found in several species, including wild turnip (Pandolfo et al. 2018), weedy rice (Presotto et al. 2024), wild carrot (Hernández et al. 2023), and wild sunflower (Hernández et al. 2024). In several cases, such admixture was associated with the origin and evolution of aggressive agricultural weeds (Vercellino, Hernández, and Presotto 2023). However, individuals resulting from interbreeding are not always successful. The success and ongoing adaptation of subsequent generations is strongly related to the environmental conditions in which they grow, the selection agents acting on them, and the fitness‐related life‐history traits that are selected upon (Exposito‐Alonso et al. 2018; Hodgins et al. 2018; Mitchell et al. 2019).

Agronomic traits selected during domestication are often offset by traits advantageous for survival in uncultivated habitats (Koziol et al. 2012; Mayrose et al. 2011; Presotto et al. 2017). While most crop‐like traits are considered ‘maladaptive’ (e.g., lack of seed dormancy, fruit retention, no branching), and reduce the fitness of crop‐wild hybrids (Mercer et al. 2006; Presotto et al. 2019), a few others can be advantageous (e.g., rapid growth, early flowering, herbicide resistance) (Mercer et al. 2007; Presotto et al. 2024), and may promote crop to wild introgression. Previous studies have also shown that the outcome of admixture largely depends on the ecological conditions experienced by hybrids (Whitton et al. 1997; Linder et al. 1998; Warwick et al. 2008; Snow et al. 2010; Pandolfo et al. 2018). However, empirical evidence showing differential fitness of crop‐wild hybrids across ecological conditions is scarce (but see Mercer et al. 2007; Presotto et al. 2019; Vercellino, Hernández, and Presotto 2023).

Agroecosystems include adjacent areas featuring agrestal environments (i.e., agricultural fields) and ruderal environments (i.e., areas which experienced disturbances and replacement of natural vegetation), such as ditches, roadsides, railways, and wire fences (Gressel 2005; Leiss and Müller‐Schärer 2001). Selection agents acting on each one are distinct (Koziol et al. 2012; Martínez‐Ghersa et al. 2000; Mayrose et al. 2011), since ruderal habitats are naturally heterogeneous in terms of biotic and abiotic conditions (e.g., moisture supply, nutrient soil fertility) while agricultural fields tend to be more homogenous due to cultivation (Altieri and Liebman 1988). Those differences may drive divergent selection, and lead to local adaptation (McAssey et al. 2016; Mitchell et al. 2021).

Trait selection varies throughout the plant life cycle and may affect the outcome of admixture. For example, selection on early life‐history traits like the timing of emergence often has cascading effects on traits expressed later in the life cycle (Huang et al. 2010; McAssey et al. 2016). Conversely, selection on advanced phenological stages may also affect traits expressed early in the offspring through maternal effects (Chiang et al. 2009; Vayda et al. 2018; Auge et al. 2019; Baskin 2023). The maternal effect, defined as the contribution of the maternal parent to the phenotype of the offspring beyond its expected chromosomal contribution (Roach and Wulff 1987), can be genetic or environmental. Here, we focused on maternal genetic effects (hereafter simply maternal effects), where phenotypic differences in the offspring are due to the maternal parent phenotype or genotype, and it is mainly attributed to the maternal provisioning of tissues, nutrients, hormones, proteins and transcripts during seed development.

Sunflower ( *Helianthus annuus* L.) is a suitable model organism for studying how gene flow between crops and wild relatives can drive the evolution of hybrids into new weeds. Cultivated sunflower originates from the common sunflower, native to North America (Crites 1993; Blackman et al. 2011), but is now widely naturalized in several countries, including Argentina, one of the world's major sunflower producers (Muller et al. 2009; Poverene et al. 2009; Mandel et al. 2011; Seiler et al. 2018; Hübner et al. 2022). Although naturalized wild populations typically grow in ruderal environments, admixture with crops can give rise to crop‐wild hybrid plants capable of invading agricultural fields and reducing crop yields (Muller et al. 2011; Casquero et al. 2013; Casquero and Cantamutto 2016). These hybrids exhibit intermediate phenotypes, combining wild‐like and crop‐like traits, with expression depending on the genetic background and direction of the cross (Hernández et al. 2017, 2021; Mercer et al. 2006). We hypothesized that the outcome of crop‐wild admixture depends on both the environment in which they grow and the direction of gene flow. To test this hypothesis, we evaluated fitness‐related traits in crop, wild and reciprocal crop‐wild hybrids over 2 years in both ruderal and agrestal environments. Specifically, we address the following questions: (i) How do ruderal and agrestal environments affect the relative fitness of crop‐wild hybrids? and (ii) Do reciprocal crop‐wild hybrids exhibit phenotypic differences driven by maternal genetic effects? This study provides insights into how admixture may shape the short‐term ecological establishment of crop–wild hybrids in novel habitats.

## 2 Material and Methods

### 2.1 Plant Material

Eight crosses (hereafter populations; Figure S1) using wild, crop and weedy *H. annuus* L. plants were performed to evaluate the influence of genetic background, environment, and maternal effects on fitness and fitness‐related traits, with a focus on wild × cultivated (crop) hybrids (“crop‐wild hybrids”). Two wild populations were collected in Argentina from ruderal habitats in Colonia Barón (BAR; 36°10′ S, 63°53′ W) and Río Cuarto (RCU; 33°09′ S, 64°20′ W), as well as a weedy feral population collected near Barrow (BRW; 38°16′ S, 60°07′ W). The cultivated parent (CROP) was an IMI‐resistant hybrid (CACIQUE CL, Criadero El Cencerro), used to produce four F1 crop‐wild hybrid populations (BARxCROP, CROPxBAR, RCUxCROP, CROPxRCU) and an F2 population of cultivated origin resembling field volunteers (VOL). The IMI‐resistant hybrid was also used to test gene transfer to crop‐wild populations by via imidazolinone herbicide application (Agrestal 2). We performed reciprocal crop‐wild hybrid populations to evaluate the maternal genetic effect on vegetative and reproductive traits.

Crosses were performed by hand‐pollination in a common garden at the Agronomy Department, Universidad Nacional del Sur, Bahía Blanca, Argentina (38°41′47″ S, 62°14′56″ W) during 2018–2019 to minimize maternal environmental effects. Populations were evaluated over the first (2019/2020) and second (2020/2021) generations derived from these hand‐pollinated plants. For each population, 10–15 female plants had several heads bagged at the pre‐flowering stage and hand‐pollinated daily with pollen from the corresponding male parent. CROP heads producing crop‐wild hybrids were emasculated each morning to prevent self‐fertilizing and pollinated in the afternoon with BAR or RCU pollen, while CROP heads for the VOL population were left to self‐fertilize. BAR and RCU heads pollinated with CROP pollen were not emasculated due to self‐incompatibility. BAR, RCU and BRW populations were produced by sibling crosses.

Pollinated heads were manually harvested and threshed at maturity. Seeds were sown at the first year (2019–2020) and, for the second year (2020–2021), seeds were obtained by crossing 15–25 plants from the same population within the same environment and sown in that environment. Further details on seed production, generations, and experimental timeline are provided in the Supporting Information (Figure S1).

### 2.2 Field Experiment

The field was plowed and leveled and had no recent history of sunflower cropping (i.e., in the last 8 years); no other sunflower seedlings germinated, except for planted populations. Populations were sown under different scenarios in which they can naturally grow, namely a ruderal environment (RUD) and two agrestal environments, wheat (AGR1) and maize (AGR2). RUD represented the natural environment where wild sunflowers grow and was recreated by allowing natural emergence and growth of species from the seed bank of the experimental soil, without removing the vegetation. Agrestal environments emulated field production in winter (AGR1) or summer (AGR2) cereal crops. Sunflower seeds were sown in early autumn (April/May) in the three environments to simulate natural dispersal, while cereal seeds from agrestals where sown later. Finally, the main differences between the environments relate to competition and availability of resources: permanent competition with spontaneous species and no external nutrient input in RUD environment (1), early competition with wheat and nutrient input in AGR1 environment (2) and late competition with maize and nutrient input in AGR2 environment (3).

The experiment was carried out as a modified split‐plot design, both years. The experimental field was divided into three main plots (125 m^2^ each plot) in which RUD, AGR1 or AGR2 management were applied. Each main plot was subdivided into five blocks (*replicates*; blocks were not ‘true replicates’ since they were not randomly assigned to but nested within each environment, which was considered for the analysis). Within the blocks, the populations were randomized into nine 0.70 m × 1.00 m plots distributed in an arrangement of 3 × 3 and separated 1 m from each other, randomly assigned to the eight populations; one plot was used as a ‘control’ to ensure no spontaneous sunflower plants emerge from the soil seed bank. Seed populations were sown in early fall (April 24, in 2019, and May 11, in 2020), with 300 seeds per plot (430 seeds/m^2^) of the corresponding population, and then covered with a thin layer of soil (~2 cm) to prevent bird predation. We planned to sow the same density both years, but seed production in the first year was highly variable between populations. Thus, we adjusted the plantings of populations that did not have 300 seeds per plot for the second year. Consequently, VOL in AGR1 and RUD, BARxCROP in RUD, and RCUxCROP in RUD and AGR1 were adjusted either by reducing the number of replicates planted, the plot area, or a combination of both, to maintain the same density as the other populations. All other populations were planted in five replicates per environment in full sized plots with the original density.

In the RUD environment, no weeds or pests were controlled and no fertilizer was added. After sowing, sunflower seedlings competed with spontaneous plant community during their complete life cycle. The plant community was similar both years and included several winter and summer annual species, such as: *Raphanus sativus* L. (most frequent in the first year), *Avena barbata* Pott., *Lolium multiflorum* L. (most frequent in the second year), *Chenopodium album* L., *Solanum elaeagnifolium* Cav., *Centaurea solstitialis* L., *Diplotaxis tenuifolia* (L.) DC., *Portulaca oleracea* L., *Portulaca grandiflora* Hook., *Salsola kali* L., *Polygonum aviculare* L., *Xanthium spinosum* L., *Datura ferox* L., *Cenchrus pauciflorus* Benth., *Tribulus terrestris* L. and *Verbesina encelioides* (Cav.) Benth. & Hook. f. ex A. Gray.

The AGR environments were managed with the techniques commonly used for each crop in the region. In AGR1, an intermediate cycle cultivar of wheat (ACA 902) was sown in the first year (June 2019), and a short cycle one (ACA 915) in the second year (August 2020). Weeds were chemically controlled before wheat planting (clodinafop‐propargyl + cloquintocet‐metyl, 30 days before, and glyphosate +2,4 D, 10 days before). Wheat density was 200 seeds m^−2^, 25 cm between the rows, both years. Inputs of monoammonium phosphate (MAP, 100 kg ha^−1^) and nitrogen (urea, 200 Kg ha^−1^; Z3 stage, Zadoks et al. 1974) were applied at sowing (MAP) and at the stem elongation stage (urea). Wheat was harvested manually in December. In AGR2, an imidazolinone (IMI) resistant maize cultivar (AX 78 CL VT3PRO, Nidera) was sown in October, both years. Chemical fallow was done with glyphosate and 2,4 D (July), and Imazetapyr herbicide was applied 30 days after sowing. It is worth noting that no hybrid plants died after the imidazolinone herbicide application, whereas wild and feral plants either died or regrew from basal nodes. Maize was sown at 6 pL m^−2^, 50 cm between the rows and 33 cm between plants in the same row, and fertilized once with MAP (130 Kg ha^−1^, 15 days after sowing), and three times with urea (225 Kg ha^−1^ each time, during the vegetative‐reproductive stages). Maize was harvest manually in April.

### 2.3 Data Collection

We measured three vegetative (pre‐flowering) and four reproductive variables each year: fall emergence, spring emergence, established plants, flowering date, reproductive plants, heads per plot and heads per plant. As sunflower seedlings usually cannot overwinter in freezing temperatures, we recorded emergence during fall–winter and spring periods aiming to estimate the non‐adaptive and adaptive germination, respectively. Emergence was recorded as seedlings per plot. Fall emergence was recorded three times in the first year (3, 6 and 9 weeks from sowing), and once (September 1) in the second year. Emergence in the second year was delayed and reduced in comparison with the first year. That is why the seedling count was delayed. Spring emergence was recorded in mid‐October, both years. Established plants per plot were counted in early summer (December 15, 2019 and January 6, 2021).

We compared the flowering of populations within the environments, aiming to identify any advancement, or delay, in flowering influenced by these factors. Thus, we compared the accumulated degree‐days from day 0 (date of first flowering plant in the experiment, each year: November 24, 2019 and December 15, 2020) to flowering date of the plot, considering a base temperature of 6.7°C (Qadir and Azim Malik 2007; Robinson 1971). Flowering date of plot was recorded twice a week, when half of the plants of the plot begun to flower (i.e., displayed at least one head with ray flowers fully extended: R5.1 stage, Schneiter and Miller 1981). Reproductive plants (i.e., those which produced at least one head with more than 10 viable fruits) and number of heads per reproductive plant were recorded when the plants completed their cycle. Overall fitness of the population was estimated by recording the number of heads produced per plot (*proxy* of fitness).

We also quantified the survival of plants in vegetative and reproductive stages. Thus, we included two variables each year: survival to establishment (i.e., the proportion of plants established in late spring relative to those emerged in spring) and survival to reproduction (i.e., the proportion of reproductive plants relative to those established).

### 2.4 Data Analysis

Statistical analysis was performed in SAS OnDemand edition. Data were analyzed using generalized linear mixed models (GLMMs) with restricted maximum likelihood in PROC GLIMMIX. We used the identity link function, transforming data prior to analysis to improve homoscedasticity when necessary. Emergence in fall–winter and spring, established plants, reproductive plants and heads per plot variables were square‐root transformed, while heads per plant was log‐transformed and flowering time was run without transformation.

#### 2.4.1 Environment and Genotype

First, we explored the environmental and genetic effects on each variable for both years. To do this, all variables were analyzed considering the environment, population and environment × population interaction as fixed effects and blocks nested within the environment as random effect (analysis I). Then, we tested genetic effects within each environment, considering population as fixed and block as random effects (analysis II). We excluded VOL from the analysis in the second year, as no plants overwintered. We also removed outliers for heads per plant analysis to improve the accuracy and simplify the model, since the number of heads largely varied with plant density (i.e., ranging from 1 to 64 plants per plot). Outliers were removed using the interquartile range (IQR) method. Differences were compared with the Tukey–Kramer test.

#### 2.4.2 Maternal Genetic Effects on Phenotypic Variation

We used orthogonal contrasts to assess phenotypic variation associated with maternal genetic effects. Specifically, we compared crop‐wild hybrids with a crop as the maternal parent (CROP×BAR and CROP×RCU) against those with a wild maternal parent (BAR×CROP and RCU×CROP) for all variables across both years.

## 3 Results

In the first year (analysis I), both the environment and population effects were significant (*p* < 0.05) for all traits, except for fall emergence which showed not significant effect of environment. The environment × population interaction was only significant for flowering time and heads per plant. Conversely, in the second year, environment, population, and the environment × population effects were significant for all variables (except the environment × population effect for heads per plot and heads per plant) (Table 1). In analysis II, the population effect was significant for all variables in all three environments for both years, except for heads per plant in AGR2 in the second year (Table 2).

**TABLE 1:** Results of analysis I from the first and second years. Linear mixed model on seven traits (transformed as described in Data Analysis) measured throughout the life cycle stages for wild, weedy, and crop‐wild hybrid populations grown in ruderal and agrestal environments from 2019 to 2021. Values for fixed factors are reported as *F*‐value_num df,denom df_ and *p*‐values; values for random factors are reported as *Z*‐values. Significant *F*‐values of fixed effects are highlighted in bold.

Factor	Effect	Fall emergence		Spring emergence		Established plants		Flowering		Reproductive plants		Heads per plot		Heads per plant	
		*F*/*Z*	*p*	*F*/*Z*	*p*	*F*/*Z*	*p*	*F*/*Z*	*p*	*F*/*Z*	*p*	*F*/*Z*	*p*	*F*_df/n_/*Z*	*p*
**YEAR 1**															
Fixed	Environment	0.5_2,12_	0.627	6.3_2,12_	**0.013**	6.3_2,12_	**0.013**	10.6_2,12_	**0.002**	4.7_2,12_	**0.031**	13.0_2,12_	**0.001**	30.6_2,12_	**< 0.0001**
Fixed	Population	54.9_7,84_	**< 0.0001**	60.8_7,84_	**< 0.0001**	21.2_7,84_	**< 0.0001**	43.9_7,75_	**< 0.0001**	20.0_7,84_	**< 0.0001**	27.2_7,84_	**< 0.0001**	35.4_7,1237_	**< 0.0001**
Fixed	Env*Pop	1.2_2,12_	0.327	1.7_14,84_	0.072	1.6_14,84_	0.095	3.2_14,75_	**0.001**	1.7_14,84_	0.072	1.8_14,84_	0.059	2.1_14,1237_	**0.009**
Random	Block (env)	1.1	0.14	1.4	0.074	1.5	0.064	0.3	0.372	1.3	0.09	0.6	0.281	1.1	0.145
Random	Residual	6.5	< 0.0001	6.5	< 0.0001	6.5	< 0.0001	6.1	< 0.0001	6.5	< 0.0001	6.5	< 0.0001	24.8	< 0.0001
**YEAR 2**															
Fixed	Environment	7.0_2,12_	**0.001**	6.9_2,12_	**0.0101**	16.0_2,12_	**0.0004**	24.6_2,12_	**< 0.0001**	18.2_2,12_	**0.0002**	18.5_2,12_	**0.0002**	22.5_2,12_	**< 0.0001**
Fixed	Population	19.4_7,76_	**< 0.0001**	29.5_7,76_	**< 0.0001**	18.7_6,66_	**< 0.0001**	6.6_6,53_	**< 0.0001**	17.9_6,66_	**< 0.0001**	10.6_6,66_	**< 0.0001**	9.0_6,640_	**< 0.0001**
Fixed	Env*Pop	2.9_14,76_	**0.0014**	3.7_14,76_	**< 0.0001**	2.3_12,66_	**0.015**	2.3_12,53_	**0.019**	2.5_12,66_	**0.01**	1.6_12,66_	0.118	2.9_12,640_	**0.0005**
Random	Block (env)	.	.	0.2	0.405	0.6	0.289	.	.	0.6	0.29	0.6	0.274	0.9	0.189
Random	Residual	6.6	< 0.0001	6.2	< 0.0001	5.7	< 0.0001	5.7	< 0.0001	5.8	< 0.0001	5.8	< 0.0001	17.8	< 0.0001

*Note:* Due to lack of plants some variables could not be measured in all replicates (blocks). Hence, where there are missing values (and data is unbalanced) the SAS Program represents it with a single decimal point (‘.’).

**TABLE 2:** Results of analysis II from the first and second years. Linear mixed models on seven traits (transformed as described in Data Analysis) measured throughout the life cycle stages for wild, weedy, and crop‐wild hybrid populations grown in ruderal and agrestal environments from 2019 to 2021. Values for fixed factors are reported as *F*‐value_num df,denom df_ and *p*‐values; values for random factors are reported as *Z*‐values. Significant *F*‐values of fixed effects are highlighted in bold.

Environment		Fall emergence		Spring emergence		Established plants		Flowering		Reproductive plants		Heads per plot		Heads per plant	
Factor	Effect	*F*/*Z*	*p*	*F*/*Z*	*p*	*F*/*Z*	*p*	*F*/*Z*	*p*	*F*/*Z*	*p*	*F*/*Z*	*p*	*F*_df/nf_/*Z*	*p*
**YEAR 1**															
AGR1 (wheat)															
Fixed	Population	29.2_7,28_	**< 0.0001**	31.2_7,28_	**< 0.0001**	5.6_7,28_	**0.0004**	4.9_7,23_	**0.002**	6.2_7,28_	**0.0002**	7.6_7,28_	**< 0.0001**	8.8_7,251_	< 0.**0001**
Random	Block	.	.	1.1	0.141	0.9	0.191	0.3	0.376	0.8	0.213	.	.	0.8	0.212
Random	Residual	4.0	< 0.0001	3.7	< 0.0001	3.7	< 0.0001	3.4	0.0004	3.7	< 0.0001	4.0	< 0.0001	11.2	< 0.0001
AGR2 (maize)															
Fixed	Population	32.4_7,28_	**< 0.0001**	21.2_7,28_	**< 0.0001**	11.1_7,28_	**< 0.0001**	38.3_7,28_	**< 0.0001**	6.0_7,28_	**0.0003**	9.0_7,28_	**< 0.0001**	20.9_7,480_	**< 0.0001**
Random	Block	0.7	0.252	0.3	0.394	.	.	.	.	.	.	0.1	0.448	.	.
Random	Residual	3.7	< 0.0001	3.7	< 0.0001	4.0	< 0.0001	4.0	< 0.0001	4.0	< 0.0001	3.7	< 0.0001	15.6	< 0.0001
RUD (ruderal)															
Fixed	Population	8.4_7,28_	**< 0.0001**	15.6_7,28_	**< 0.0001**	7.6_7,28_	**< 0.0001**	34.5_7,28_	**< 0.0001**	10.5_7,28_	**< 0.0001**	25.2_7,28_	**< 0.0001**	13.8_7,506_	**< 0.0001**
Random	Block	0.9	0.194	0.9	0.19	1.0	0.15	0.8	0.21	1.0	0.157	1.0	0.159	1.1	0.147
Random	Residual	3.7	< 0.0001	3.7	< 0.0001	3.7	< 0.0001	3.5	0.0003	3.7	< 0.0001	3.7	< 0.0001	15.9	< 0.0001
**YEAR 2**															
AGR1 (wheat)															
Fixed	Population	10.2_7,24_	**< 0.0001**	14.7_7,24_	**< 0.0001**	11.5_6,22_	**< 0.0001**	1.6_6,22_	0.205	10.8_6,22_	**< 0.0001**	7.0_6,22_	**0.0003**	12.2_6,337_	**< 0.0001**
Random	Block	.	.	.	.	.	.	.	.	.	.	.	.	0.5	0.314
Random	Residual	3.7	< 0.0001	3.7	0.049	3.6	0.0002	3.6	0.0002	3.6	0.0002	3.6	0.0002	13.0	< 0.0001
AGR2 (maize)															
Fixed	Population	4.4_7,28_	**0.0023**	6.5_7,28_	**0.0001**	6.9_6,24_	**0.0003**	4.1_6,17_	**0.01**	6.4_6,24_	**0.0004**	3.5_6,24_	**0.013**	5.7_6,166_	**< 0.0001**
Random	Block	.	.	0.4	0.337	0.1	0.49	0.8	0.207	0.1	0.483	0.4	0.353	0.7	0.234
Random	Residual	4.0	< 0.0001	3.7	< 0.0001	3.5	0.0003	2.9	0.0016	3.5	0.0003	3.5	0.0003	9.1	< 0.0001
RUD (ruderal)															
Fixed	Population	13.8_7,24_	**< 0.0001**	23.0_7,24_	**< 0.0001**	6.9_6,20_	**0.0004**	27.5_6,14_	**< 0.0001**	7.5_6,20_	**0.0003**	10.3_6,20_	**< 0.0001**	1.5_6,137_	0.172
Random	Block	0.3	0.395	0.1	0.464	0.9	0.193	.	.	0.9	0.184	0.6	0.278	0.4	0.355
Random	Residual	3.5	0.0003	3.5	0.0002	3.2	0.0008	3.0	0.0013	3.2	0.0003	3.2	0.0008	7.9	< 0.0001

*Note:* Due to lack of plants some variables could not be measured in all replicates (blocks). Hence, when there are missing values (and data is unbalanced) the SAS Program represents it with a single decimal point (‘.’).

**FIGURE 1:**
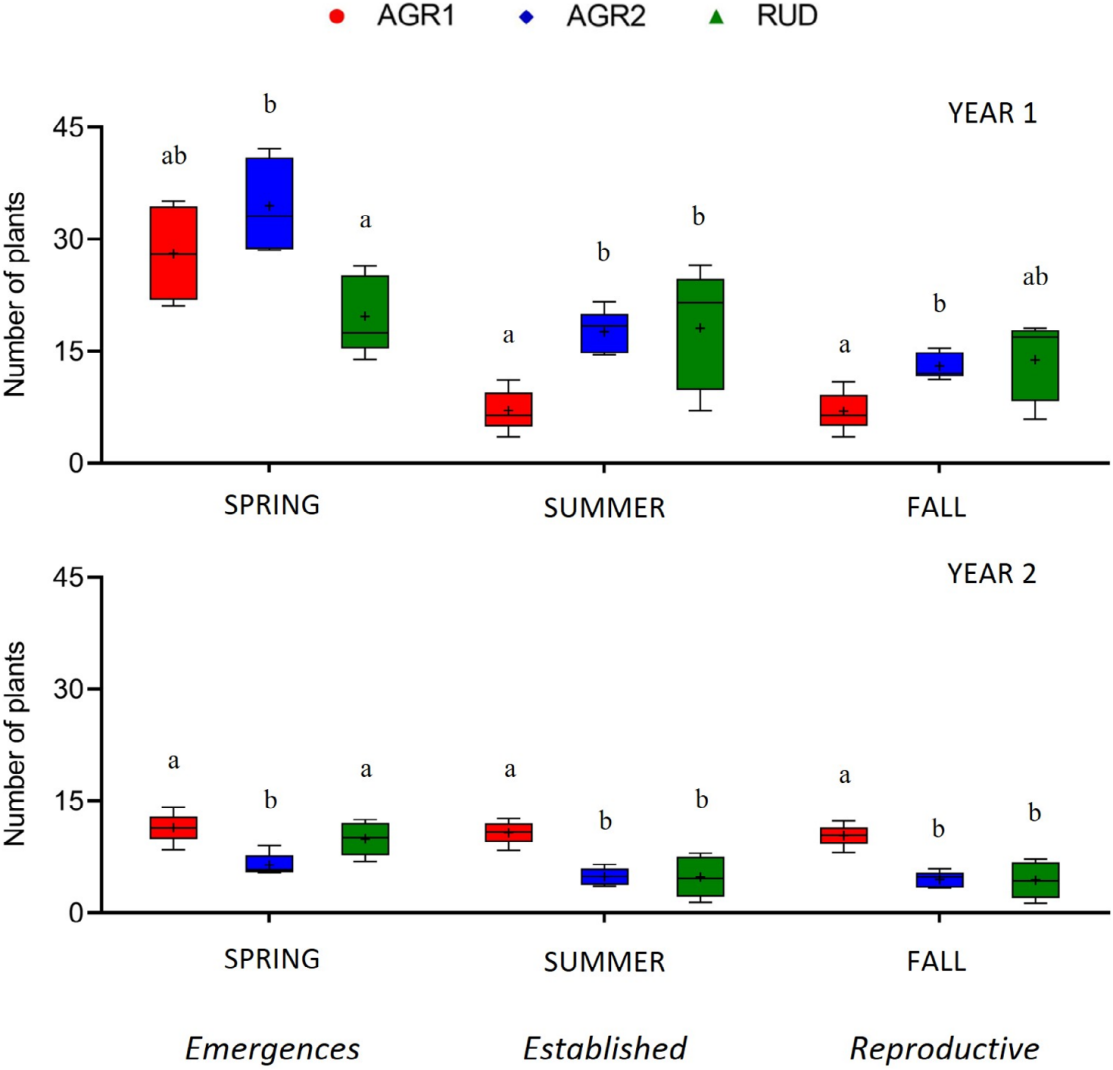
Number of plants during different life‐history stages in the first year (above) and second year (below) in wheat (AGR1), maize (AGR2) and ruderal (RUD) environments: Emergence in spring (SPRING), established plants (SUMMER), and reproductive plants (FALL). Backtransformed least square means are indicated by the + symbol, and those sharing a letter within the same season and year are not significant by Tukey's test.

High levels of off‐season germination were observed in the first year in all three environments. At 6 weeks, average seedlings per plot were 80.8 ± 1.6 (AGR1), 88.4 ± 5.1 (AGR2) and 77.6 ± 8.4 (RUD), representing 25.9%–29.5% of sown seeds. Populations also differed within environments: BAR, BARxCROP, RCUxCROP, and RCU generally showed the highest fall emergence (except RCU in RUD; Table S3, Figure S2). By week nine, all seedlings died due to unusually low winter temperatures (−7.5°C to −3.6°C; Figure S3). Consequently, spring emergence arose from a new, much smaller cohort, except for BRW, which showed higher spring than fall emergence. Spring emergence was greater in AGR2 than in AGR1 and RUD, with consistent populations differences: BRW highest, VOL, BARxCROP and RCUxCROP lowest, and the rest intermediate (Table S3). In the second year, fall emergence was delayed and reduced, with significantly lower values in AGR2 (3.8 ± 0.7 seedlings per plot), than in AGR1 (6.3 ± 0.5) and RUD (7.1 ± 0.7). Spring emergence included fall seedlings and remained higher in AGR1 and RUD than in AGR2 (Figure 1). BAR, RCU and BRW had the greatest numbers across environments (except RCUxCROP in AGR1), while crop‐wild hybrids showed similar emergence regardless of environment (Table S4). VOL emergence was negligible everywhere, and no plants were established (Table S4).

Plant density (established and reproductive plants) was influenced by environment and population in the first year, and by their interaction in the second (Table 1). Established and reproductive plant numbers were higher in AGR2 and RUD than in AGR1 in the first year, but this pattern reversed in the second (Figure 1). Across environments and years, populations generally showed similar numbers of established and reproductive plants, although feral BRW and wild BAR and RCU consistently produced more reproductive plants than RCUxCROP, BARxCROP, and VOL; CROPxBAR and CROPxRCU resembled feral and wild populations in the first year but aligned with reciprocal hybrids in the second (Tables S3 and S4). Survival to establishment also differed between environments in both years, with population effects evident only in AGR2 in the first year. Survival was higher in RUD than in AGR environments in the first year, but the opposite occurred in the second (Figure 1, Table S2). Overall, survival to the reproductive stage was high in AGR environments, while in RUD it was lower in the first year but similar to AGR in the second (Figure 1). Population‐level differences were observed in AGR2 and RUD during the first year, and only in RUD in the second: BRW showed higher mortality in AGR2, while VOL and RCUxCROP had the highest mortality in RUD in the first and second year, respectively (Tables S1 and S2).

**FIGURE 2:**
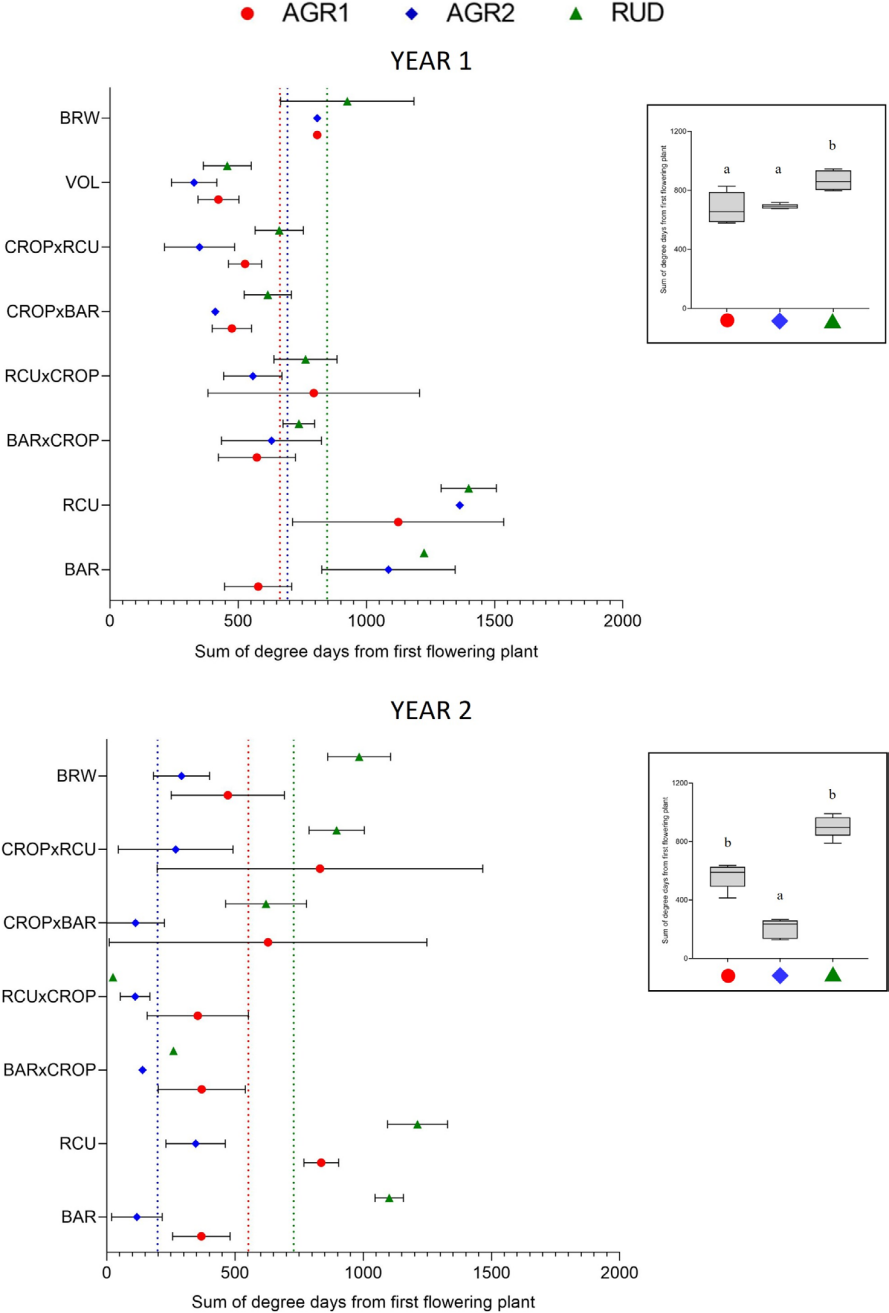
Flowering time (least square mean ± standard deviation) between populations for each year (2019–2020, above; 2020–2021, below) and environment: AGR1 (red circles), AGR2 (blue diamonds) and RUD (green triangles). Flowering time is measured in days since the first head of the field season opened (regardless of plot; November 24, 2019, and December 16, 2020) until half of the plants in a plot reached the flowering stage. Means by environment are represented by dotted lines (on the left) and the panel (on the right) for each year.

Flowering occurred earlier in the agrestal than ruderal environments both years, with populations differing significantly within each environment, except in AGR1 during the second year (Figure 2, Tables 1 and 2). Differences between populations were lower in AGR1 than in other environments, although flowering was highly variable between plants from the same population. In general, VOL and the four crop‐wild hybrids flowered earlier, followed by BRW, and then wilds, in all environments in the first year, while in the second year, we observed the same pattern in RUD, but it was less evident in AGR1 and AGR2. Also, wild BAR always flowered earlier than wild RCU (Tables S3 and S4).

**FIGURE 3:**
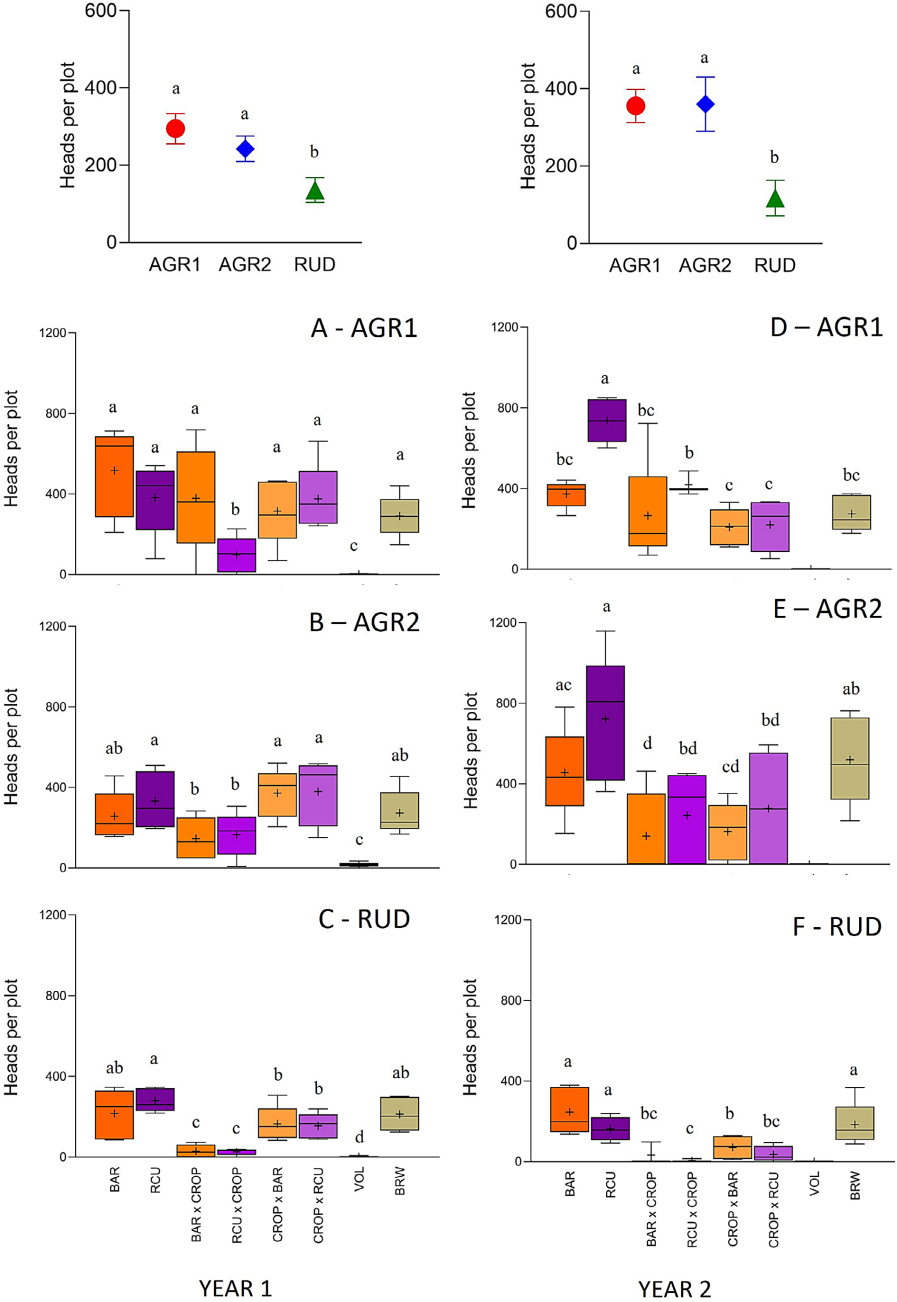
Mean heads per plot during the first (left) and second (right) year, by environment (above) and by population and environment interaction (below): AGR1 (A), AGR2 (B), and RUD (C), in the first year, and AGR1 (D), AGR2 (E), and RUD (F), in the second year. The box represents the range between the first and third quartiles, with the median indicated by the line inside the box. Whiskers extend from the minimum to the maximum values, excluding outliers. Backtransformed least square means are indicated by the + symbol. Statistical significance between environments is indicated by **p < 0.01 and ***p < 0.001. Means sharing letters are not significantly different (*p* < 0.05) according to Tukey–Kramer's tests.

The overall fitness of plants was higher in AGR than in RUD environments in both years (Figure 3). VOL consistently showed the lowest fitness across environments and years. In the first year, within AGR1, most populations produced a similar number of heads per plot (290.4–516.2), except for RCUxCROP, which produced only 96.8 heads (Figure 3A). In AGR2, wild, feral, and crop‐female parent hybrids produced more heads (256.8–379.4) than wild‐female parent hybrids (145.8–165). RCU performed similarly to crop‐female parent hybrids, while BAR and feral BRW were intermediate between the two groups (Figure 3B). In the second year, in AGR1, crop‐wild hybrids performed similarly to BAR and BRW (209–418.7) but were less fit than RCU (736.2). In AGR2, RCU again outperformed all other populations (722.6), while crop‐wild hybrids were at the opposite extreme (140.4–276.8), although most did not differ significantly from BAR or BRW (455.8–519.2; Figure 3D,E). In contrast, in RUD environments, crop‐wild hybrids consistently produced fewer heads than wild and feral populations. In the first year, BAR, RCU and BRW performed significantly better (212.6–279.8) than wild‐female parent hybrids (25.6–29.4), while crop‐female parent hybrids performed at an intermediate level between the two groups (154.8–164‐6). Moreover, in the second year, wild and feral populations performed significantly better (162.6–246‐6) than all crop‐wild hybrids (5–71.2; Figure 3C,F).

Consistent with the number of reproductive plants per plot, the average number of heads per plant varied across environments, even when overall fitness was similar. In the first year, plants produced more heads in AGR1 (35.5 ± 2.8) than in AGR2 (15.9 ± 0.8), while RUD showed the lowest values (8.1 ± 0.3). VOL consistently had the fewest heads, followed by BRW, whereas BAR and wild‐female parent hybrids outperformed RCU and crop‐female parent hybrids in both AGR environments. In RUD, values were more uniform, except for VOL and BRW. In the second year, averages were highest in AGR2 (70.8 ± 4.8), followed by AGR1 (31.6 ± 1.9) and RUD (23.1 ± 1.9). BRW again produced fewer heads (VOL did not survive), while crop‐wild hybrids generally exceeded wild plants in AGR environments. In RUD, RCUxCROP showed the lowest values, whereas BAR, RCU, and the other hybrids ranged from 15.6 to 44.5 heads, with no significant differences between populations (Tables S3 and S4).

We found phenotypic differences between reciprocal hybrids consistent with maternal genetic effects across all environments in the first year, and only in AGR1 in the second year (Table 3). In the first year, these differences were similar across environments: wild‐female parent hybrids produced more seedlings in fall–winter, whereas crop‐female parent hybrids produced more in spring, a pattern that persisted through later stages (established and reproductive plants). Crop‐female parent hybrids also flowered earlier and produced more heads per plot, while wild‐female parent hybrids produced more heads per plant in AGR but not in RUD environments. In the second year, in AGR1, wild‐female parent hybrids consistently produced more plants from fall emergence to the end of the cycle, but fewer heads per plant, resulting in similar overall fitness (heads per plot) compared with crop‐female parent hybrids.

**TABLE 3:** Maternal genetic effects on seven traits (backtransformed least square mean values ± standard error) measured throughout the life cycle stages for reciprocal sunflower crop‐wild hybrids (CROPxWILD vs. WILDxCROP) during the first and second generations (year 1 and 2, respectively) under agrestal (wheat: AGR1 and maize: AGR2) and ruderal (RUD) environments. ‐Fvalues are for 1 df contrasts.

Traits		AGR1			AGR2			RUD		
		CROPxWILD	WILDxCROP	*F*	CROPxWILD	WILDxCROP	*F*	CROPxWILD	WILDxCROP	*F*
Year 1	Fall emergence	25.00 (± 4.74)	166.70 (± 13.28)	**154.73** ***	31.50 (± 6.17)	187.60 (± 11.40)	**172.84** ***	49.90 (± 13.57)	135.70 (± 13.24)	**31.21** ***
	Spring emergence	23.20 (± 5.03)	8.20 (± 2.02)	**18.75** ***	23.60 (± 4.19)	14.10 (± 2.27)	3.01 ns	17.50 (± 3.12)	4.40 (± 0.82)	**13.13** ***
	Established	10.40 (± 1.48)	2.80 (± 1.00)	**15.35** ***	18.50 (± 3.28)	4.90 (± 0.80)	**14.65** ***	16.90 (± 3.17)	4.70 (± 1.65)	**11.11** **
	Flowering (GD)	501.21 (± 22.75)	684.17 (± 109.95)	2.84 ns	380.24 (± 30.57)	593.77 (± 49.07)	**12.59** **	637.93 (± 28.72)	751.66 (± 35.94)	2.86 ns
	Reproductive	10.20 (± 1.35)	2.80 (± 0.96)	**15.42** ***	16.50 (± 2.88)	4.90 (± 0.77)	**13.53** ***	13.70 (± 2.42)	3.40 (± 0.95)	**14.24** ***
	Heads per plot	345.60 (± 50.20)	237.50 (± 75.28)	**4.44** *	375.50 (± 42.80)	155.40 (± 31.98)	**18.18** ***	159.70 (± 22.72)	27.50 (± 7.54)	**46.65** ***
	Heads per plant	28.36 (± 2.51)	75.70 (± 14.73)	**11.65** ***	20.77 (± 1.52)	29.94 (± 3.92)	**4.27** *	10.13 (± 0.54)	7.48 (± 0.94)	**4.45** *
Year 2	Fall emergence	2.40 (± 0.60)	5.88 (± 2.41)	**7.75** *	1.90 (± 0.66)	1.40 (± 0.62)	0.15 ns	2.20 (± 0.74)	3.33 (± 0.84)	0.54 ns
	Spring emergence	4.60 (± 1.15)	9.88 (± 3.29)	**6.16** *	3.00 (± 1.29)	1.70 (± 0.58)	0.05 ns	2.70 (± 0.65)	4.50 (± 1.48)	2.21 ns
	Established	3.80 (± 0.79)	9.38 (± 3.27)	**9.37** **	2.30 (± 0.94)	1.50 (± 0.54)	0 ns	1.40 (± 0.40)	1.17 (± 0.82)	0.88 ns
	Flowering (GD)	730.14 (± 189.96)	364.82 (± 58.81)	5.26 ns	179.32 (± 65.55)	120.42 (± 19.23)	0.22 ns	724.49 (± 68.77)	142.40 (± 118.2)†	**15.18** ***
	Reproductive	3.80 (± 0.79)	9.25 (± 3.09)	**7.01** *	2.20 (± 0.87)	1.50 (± 0.56)	0.06 ns	1.40 (± 0.40)	1.00 (± 0.82)	0.32 ns
	Heads per plot	214.50 (± 33.29)	323.00 (± 22.28)	0.73 ns	219.50 (± 68.86)	192.00 (± 67.08)	0.04 ns	49.70 (± 16.14)	18.83 (± 16.02) †	1.35 ns
	Heads per plant	56.44 (± 7.73)	28.44 (± 2.60)	**13.09** ***	99.77 (± 19.40)	128.00 (± 15.85)	1.7 ns	35.50 (± 10.38)	18.83 (± 4.59) †	0.22 ns

*Note:* Significant *F*‐values are highlighted in bold. Means with † indicate data from only two plots with surviving plants.

Abbreviation: ns, Non‐significant.

*** *p* < 0.001.

** *p* < 0.01.

* *p* < 0.05.

## 4 Discussion

This study provides an ecological assessment of early crop‐wild hybrid establishment under realistic field conditions, examining the interaction between environmental and genetic factors throughout the plant life cycle. We found that the fitness of crop‐wild hybrids is strongly influenced by environment, maternal genetic effects and their interactions, indicating that complex interactions determine the success or failure of hybrids in the field. Overall, we observed that agrestal environments are more favorable than ruderal ones for the establishment and persistence of crop‐wild hybrids, which is in line with previous studies in sunflower (Mercer et al. 2007; Muller et al. 2011; Casquero et al. 2013; Hernández et al. 2022) but also in other crop complexes (but see Vercellino, Hernández, Pandolfo, et al. 2023 for a review). Interestingly, other species within the genus *Helianthus* have also become invasive as a result of admixture (e.g., *H. tuberosus* in Europe; Bock et al. 2018) or interspecific hybridization ( *H. petiolaris* in Argentina; Mondon et al. 2018). We also found maternal effects on early life history traits, with cascading effects on traits expressed later in the life cycle, including fitness. However, maternal effects were only observed during the first year and were not in the expected direction, highlighting their importance immediately after gene flow but also their limited impact on longer‐term evolutionary processes.

### 4.1 Influence of Environment Across the Life Cycle in Wild and Hybrid Sunflower Populations

Selection on early life‐history traits may affect traits expressed later in the life cycle (Huang et al. 2010). Here, for crop‐wild hybrids, we observed high out‐of‐season emergence in the first year, likely as a result of the reduced level of dormancy inherited from the cultivated parent (Snow et al. 1998; Pace et al. 2015; Hernández et al. 2017). The increased out‐of‐season emergence resulted in low overwinter survival and fitness (Hernández et al. 2019, 2021). However, over the second year, out‐of‐season emergence was limited, likely as a consequence of the strong negative selection on this trait observed during the first year. These results are important as standard phenotypic selection experiments utilized to predict the outcomes of gene flow only span a single season (Lande and Arnold 1983; Kingsolver et al. 2001; Siepielski et al. 2013; Svensson and Kvarnemo 2023), missing the contribution of negative selection of maladaptive traits (e.g., out‐of‐season emergence in this case) immediately after admixture. However, because we only evaluated 2 years, we cannot tease apart the role of selection during the first year and the interannual environmental variability in phenotypic expression.

On the other hand, the differences observed in overall emergence between agrestal and ruderal environments in the first year (higher in AGR environments) may be attributed to alterations caused by crop management (e.g., fallow, sowing), or an increased nutrient and water availability in AGR environments due to fertilization and weed control (Leiss and Müller‐Schärer 2001; Presotto et al. 2020). In addition, in the ruderal environment, spontaneous species provide cover over the year, resulting in a low red‐to‐far‐red light ratio, which is known to negatively affect seed germination and emergence in sunflower (Huarte and Arnold 2003).

Flowering time is influenced by the environmental abiotic factors and is typically under strong selection during the colonizing of novel habitats (Chamorro and Sans 2010; Debieu et al. 2013; Hovick et al. 2012; Mercer et al. 2006; Palacio‐Lopez et al. 2020). In our experiments, flowering was earlier in agrestal environments compared with the ruderal ones, and in crop‐wild hybrids compared with their wild counterparts. In agricultural habitats, frequent disturbances are expected to favor early flowering and shorter life cycles, while ruderal habitats tend to delay growth, biomass accumulation, and flowering (Chamorro and Sans 2010; Vercellino, Hernández, Pandolfo, et al. 2023). Although our results are in line with this expectation, we did not track the cycle of individual plants; therefore, it is possible that late flowering is a result of later emergence than genetic differences in flowering time. We also found that hybrids flowered earlier than their wild counterparts, likely as result of early flowering alleles carried by cultivars (Burke et al. 2002; Presotto et al. 2011). However, due to our experimental design, we cannot tease apart genetically based differences from plasticity. Further studies comparing hybrids selected under distinct habitats (e.g., agrestal vs. ruderal) in a common garden are needed to evaluate the relative contribution of genetically based differences in flowering time (and other traits) and plasticity on the adaptation to specific habitats.

When crops and wild relatives interbreed, crop‐wild hybrids often show lower fitness than their wild parents, limiting the introgression of cultivar alleles (Snow et al. 2001; Spencer and Snow 2001; Liu 2021; Kanatas et al. 2021; Vercellino, Hernández, and Presotto 2023; Zhang et al. 2023). However, the relative fitness of crop‐wild hybrids varies with environmental conditions (Campbell and Snow 2007; Hovick et al. 2012; Mercer et al. 2006, 2007). Here, we found that crop‐wild hybrids exhibited lower fitness than wild populations in ruderal environments. However, in agrestal environments ―particularly under wheat competition― the relative fitness of hybrids was higher, suggesting that these conditions are more favorable for crop‐wild hybrids. In general, the increased relative fitness was associated with the plants' ability to produce more heads rather than with enhanced survival or reproductive success. This plasticity in the number of heads per plant in response to density is strongly associated with branching (Presotto et al. 2019), and may play a key role in hybrid populations in recovering fitness following strong negative selection during establishment.

### 4.2 Maternal Genetic Effect in Crop‐Wild Hybrids

Maternal effects can influence the outcome of hybridization by controlling the expression of early life history traits, like seed germination and seedling emergence (Roach and Wulff 1987; Hernández et al. 2021). During the first year, we observed phenotypic differences between reciprocal hybrids, consistent with maternal effects. Crop‐female parent hybrids showed lower off‐season emergence than their reciprocal counterparts, resulting in more established plants and higher fitness. Although this is opposite to our expectations (Hernández et al. 2017, 2021), weedy biotypes of sunflower with crop‐female parents have repeatedly evolved in Europe (Muller et al. 2011) and Argentina (Casquero et al. 2013; Hernández et al. 2022), indicating that maladaptive traits inherited from cultivated parents can be important immediately after admixture, but have little impact on evolutionary processes. Supporting this, we did not observe any maternal effect for fitness in the second year. Our results suggest that a strong initial selection for maladaptive traits can facilitate introgression in crop maternal parent hybrids. Further studies comparing post‐selection reciprocal crop‐wild hybrids in a common garden will be useful to investigate the genetic signatures of that strong initial negative selection.

### 4.3 Limitations and Future Directions

This study was conducted over 2 years on crop‐wild hybrids grown in two agrestal and one ruderal environment, and provides important insights into the flexibility of ecological barriers of crop‐to‐wild introgression. Although previous studies have shown rapid evolution of crop‐wild hybrids ―attributed to gene linkage and the persistence of large segments of intact chromosomes transferred between wild and cultivated forms― this timeframe may be insufficient to reliably infer their evolutionary trajectories (Whitton et al. 1997; Campbell et al. 2009; Presotto et al. 2016), and thus the results should be interpreted with caution. Further studies could include reciprocal transplant trials to infer adaptation to these environments, which, when combine with genomic tools, could provide a more integrative and deeper understanding of adaptive processes (Johnson et al. 2022). Although we did not use molecular markers to confirm admixture, a subset of hybrid plants was exposed and survived a lethal dose of Imazethapyr, while non‐hybrids either died or regrew from basal nodes with obvious symptoms of herbicide damage. This confirms that all putative hybrids carry one copy of the IMI‐resistant gene from the crop. Finally, this experiment was carried out in one field site where winter spontaneous species are predominant and under specific crop sequences associated with the predominant winter and summer species cultivated in Argentina. To extend these results, future experiments should be carried out varying field sites (e.g., more humid) and crop rotations (e.g., barley, sunflower or soybean).

## 5 Conclusion

Our study shows that ecological barriers for selection vary with the environment and maternal genetic effects, being weaker for agrestal than ruderal environments, and for crop female parent than wild female parent hybrids. This helps to explain both the overrepresentation of crop‐wild hybrids among agricultural weed species (Vercellino, Hernández, Pandolfo, et al. 2023) and the repeated origin of weedy sunflowers with crop female parents in Europe (Muller et al. 2011) and Argentina (Casquero et al. 2013; Hernández et al. 2022). Our result also highlights the need to scrutinize natural crop‐wild hybrid zones to prevent the origin of hybrid populations.

## Author Contributions

**Ignacio J. Fanna:** data curation (lead), formal analysis (lead), investigation (lead), methodology (lead), supervision (equal), writing – original draft (lead), writing – review and editing (equal). **Fernando Hernández:** conceptualization (equal), formal analysis (supporting), methodology (equal), writing – review and editing (equal). **Kristin L. Mercer:** conceptualization (equal), formal analysis (supporting), methodology (supporting), writing – review and editing (equal). **Alejandro Presotto:** conceptualization (equal), formal analysis (supporting), methodology (equal), resources (lead), writing – review and editing (equal).

## Funding

This work was supported by Agencia Nacional de Promoción de la Investigación, el Desarrollo Tecnológico y la Innovación (Grant PICT 2017‐0473), Universidad Nacional del Sur (PGI 24/A244), and the Ohio Agricultural Research and Development Center.

## Conflicts of Interest

The authors declare no conflicts of interest.

## Supporting information


**Data S1:** ece372961‐sup‐0001‐supinfo.docx.

## Data Availability

The data that supports the findings of this study is openly available in Figshare https://doi.org/10.6084/m9.figshare.29120186.
