# Supplementary material for: Agrestal Environments and Maternal Genetic Effects Weaken the Ecological Barriers for Crop to Wild Introgression in Sunflower
Source: Ecol Evol. 2026 Feb 3;16(2):e72961. doi: 10.1002/ece3.72961 (PMC12865504; doi:10.1002/ece3.72961)
Supplement: Supplementary file 1 — Data S1: ece372961‐sup‐0001‐supinfo.docx. [file ECE3-16-e72961-s001.docx]

**Agrestal environments and maternal genetic effects weaken the ecological barriers for crop to wild introgression in sunflower**

*Journal of Plant Research.* Subject: *Ecology/Ecophysiology/Environmental biology*

Ignacio J. Fanna^1^, Fernando Hernández^2^, Kristin L. Mercer^3,4^, Alejandro Presotto^1^

^1^Departamento de Agronomía, CERZOS, Universidad Nacional del Sur (UNS)-CONICET, San Andrés 800, 8000, Bahía Blanca, Argentina

^2^Department of Botany and Biodiversity Research Centre, University of British Columbia, Vancouver, British Columbia, Canada

^3^Department of Horticulture and Crop Science, The Ohio State University, Columbus, Ohio, United States of America

^4^Institute of Sustainable Agroecosystem Services, University of Tokyo, Tokyo, Japan

Corresponding authors:

1. Kristin L. Mercer, Department of Horticulture and Crop Science, The Ohio State University, Columbus, Ohio, United States of America. E- mail: mercer.97@osu.edu


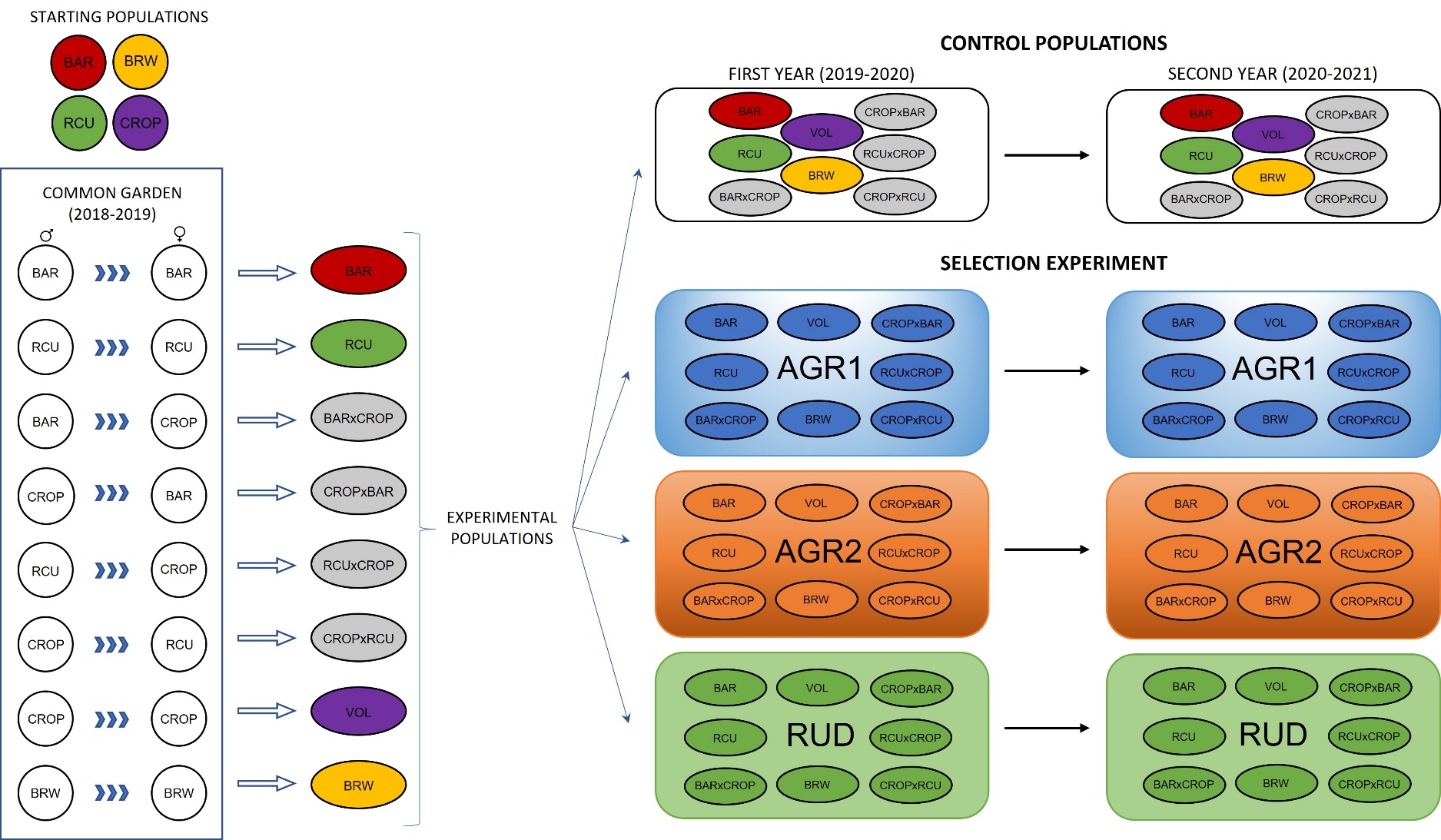


Figure S1. Scheme of crosses and timeline of experiments with populations used in each one. On the left: populations produced from crosses in the common garden. On the right: the eight populations sown in RUD, AGR1 and AGR2 environments and multiplied within the same environment for the second year.


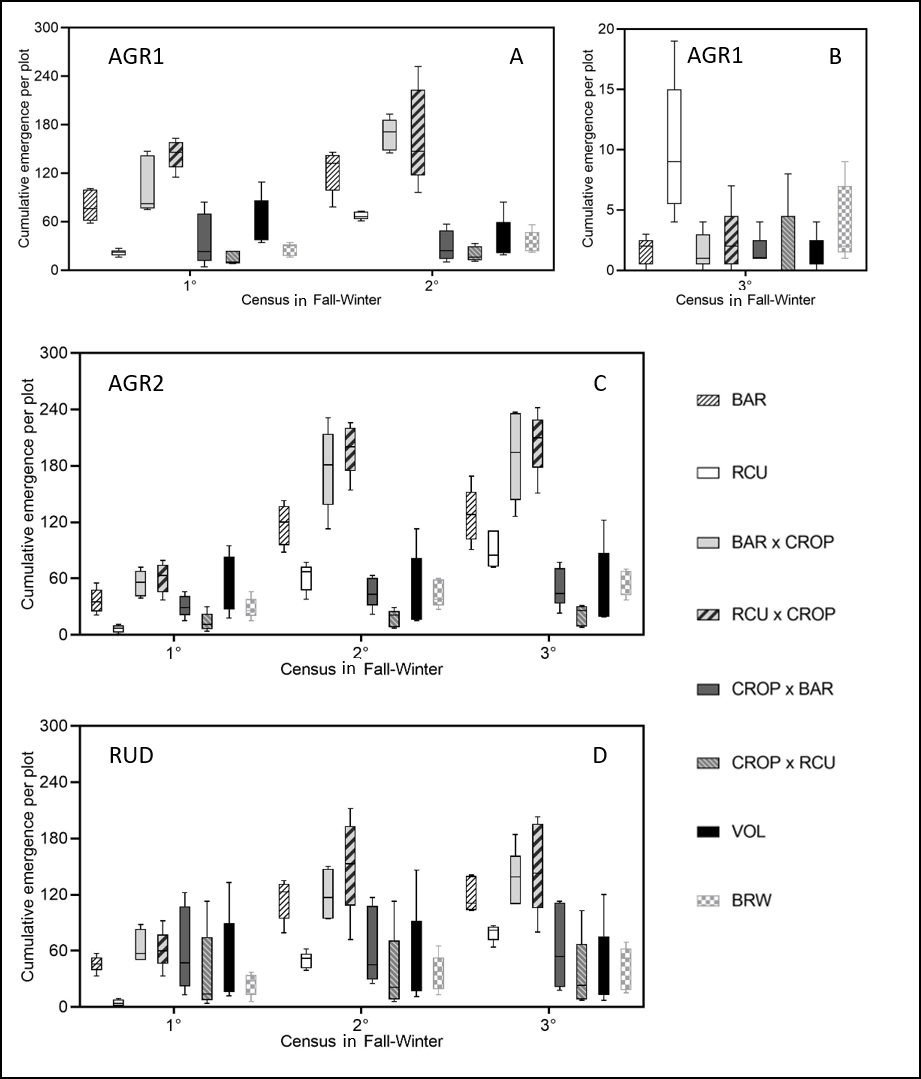


Figure S2. Cumulative emergence of seedlings during censuses in fall-winter, 2019. (A) Seedlings in the first and second census in AGR1. (B) Seedlings in the third census in AGR1. (C) Seedlings in the census in AGR2. (D) Seedlings in the census in RUD. No significant differences were found between environments within the same period, except in the third census, when AGR1 had few emergences due to recent herbicide application.

###
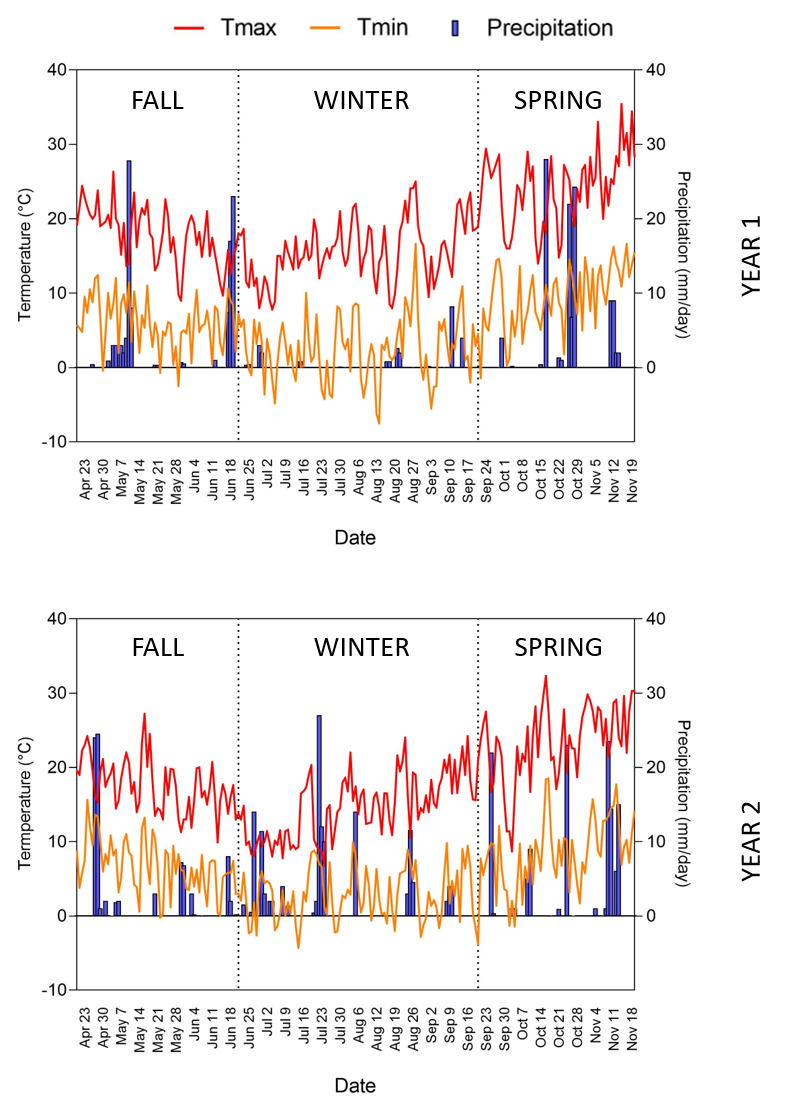


Figure S3. Daily weather conditions during 2019 and 2020 years in Bahía Blanca (Buenos Aires). Temperature (°C; maximum: red, minimum: orange) on the left axis, rainfall (mm.day^-1^; blue) on the right axis. Data obtained from CCT-Bahía Blanca meteorological station.

Table S1. ANOVA results of the analysis of survival to establishment (proportion of established plants compared to those that emerged in spring), and survival to reproduction (proportion of reproductive plants compared to those established) each year. Environment, population and interaction were considered as fixed and block as random effects, in analysis I. Population (environment) was considered as fixed and block as random effects, in analysis II. p-value: **P < 0.001; *P < 0.01; NS, not significant.

| YEAR 1: VEGETATIVE STAGE | | | | YEAR 1: REPRODUCTIVE STAGE | | | |
| --- | --- | --- | --- | --- | --- | --- | --- |
| **Analysis I** | *p-*value | **Analysis II** | *p-*value | **Analysis I** | *p-*value | **Analysis II** | *p-*value |
| Environment | ** | AGR1 | NS | Environment | * | AGR1 | NS |
| Population | NS | AGR2 | * | Population | ** | AGR2 | ** |
| Env*Population | NS | RUD | NS | Env*Population | ** | RUD | ** |
| YEAR 2: VEGETATIVE STAGE | | | | YEAR 2: REPRODUCTIVE STAGE | | | |
| **Analysis I** | *p-*value | **Analysis II** | *p-*value | **Analysis I** | *p-*value | **Analysis II** | *p-*value |
| Environment | ** | AGR1 | NS | Environment | NS | AGR1 | NS |
| Population | NS | AGR2 | NS | Population | ** | AGR2 | NS |
| Env*Population | NS | RUD | NS | Env*Population | * | RUD | ** |

Table S2. Proportion of plants at the vegetative (VE) and reproductive stage (RE) and year (i.e., VE: established plants/emergence in spring; RE: reproductive plants/established plants). Means (± SE) with different letters within the same stage are significantly different (P < 0.05) by Tukey-Kramer’s test.

| Environment, population | | YEAR 1 | | YEAR 2 | |
| --- | --- | --- | --- | --- | --- |
|  |  | VE | RE | VE | RE |
| AGR1 (1) | | 0.35 (± 0.10) ^a^ | 0.96 (± 0.03) ^b^ | 0.95 (± 0.07) ^b^ | 0.98 (± 0.03) ^a^ |
| AGR2 (2) | | 0.57 (± 0.10) ^b^ | 0.87 (± 0.03) ^b^ | 0.83 (± 0.07) ^b^ | 1.00 (± 0.03) ^a^ |
| RUD (3) | | 1.11 (± 0.10) ^b^ | 0.75 (± 0.03) ^a^ | 0.47 (± 0.07) ^a^ | 0.90 (± 0.04) ^a^ |
| (1) | BAR | 0.24 (± 0.27) ^a^ | 1.02 (± 0.08) ^ab^ | 0.98 (± 0.13) ^a^ | 1.00 (± 0.07) ^a^ |
|  | RCU | 0.21 (± 0.27) ^a^ | 1.00 (± 0.08) ^ab^ | 1.00 (± 0.13) ^a^ | 0.94 (± 0.07) ^a^ |
|  | BARxCROP | 0.51 (± 0.27) ^a^ | 1.02 (± 0.09) ^ab^ | 1.08 (± 0.13) ^a^ | 1.00 (± 0.07) ^a^ |
|  | RCUxCROP | 0.49 (± 0.27) ^a^ | 1.00 (± 0.09) ^ab^ | 0.86 (± 0.17) ^a^ | 1.00 (± 0.07) ^a^ |
|  | CROPxBAR | 0.64 (± 0.27) ^a^ | 1.16 (± 0.08) ^a^ | 0.93 (± 0.13) ^a^ | 1.00 (± 0.07) ^a^ |
|  | CROPxRCU | 0.44 (± 0.27) ^a^ | 1.00 (± 0.08) ^ab^ | 0.90 (± 0.13) ^a^ | 1.00 (± 0.07) ^a^ |
|  | VOL | 0.12 (± 0.27) ^a^ | 0.50 (± 0.10) ^b^ | - | - |
|  | BRW | 0.14 (± 0.27) ^a^ | 0.98 (± 0.08) ^ab^ | 0.94 (± 0.13) ^a^ | 0.94 (± 0.07) ^a^ |
| (2) | BAR | 0.68 (± 0.27) ^ab^ | 0.68 (± 0.08) ^bc^ | 0.88 (± 0.13) ^a^ | 1.00 (± 0.07) ^a^ |
|  | RCU | 0.50 (± 0.27) ^ab^ | 0.82 (± 0.08) ^abc^ | 0.84 (± 0.13) ^a^ | 0.93 (± 0.07) ^ab^ |
|  | BARxCROP | 0.30 (± 0.27) ^b^ | 1.08 (± 0.08) ^a^ | 0.62 (± 0.21) ^a^ | 1.50 (± 0.11) ^ab^ |
|  | RCUxCROP | 0.52 (± 0.27) ^ab^ | 0.95 (± 0.08) ^ab^ | 0.99 (± 0.15) ^a^ | 0.75 (± 0.08) ^b^ |
|  | CROPxBAR | 0.81 (± 0.27) ^a^ | 0.93 (± 0.08) ^ab^ | 0.88 (± 0.15) ^a^ | 1.00 (± 0.08) ^ab^ |
|  | CROPxRCU | 0.70 (± 0.27) ^ab^ | 0.91 (± 0.08) ^ab^ | 0.80 (± 0.17) ^a^ | 0.96 (± 0.09) ^ab^ |
|  | VOL | 0.65 (± 0.27) ^ab^ | 1.02 (± 0.08) ^a^ | - | - |
|  | BRW | 0.42 (± 0.27) ^ab^ | 0.56 (± 0.08) ^c^ | 0.80 (± 0.13) ^a^ | 0.91 (± 0.07) ^ab^ |
| (3) | BAR | 2.20 (± 0.27) ^a^ | 1.00 (± 0.08) ^a^ | 0.66 (± 0.13) ^a^ | 0.96 (± 0.07) ^a^ |
|  | RCU | 1.28 (± 0.27) ^a^ | 0.86 (± 0.08) ^a^ | 0.68 (± 0.13) ^a^ | 0.95 (± 0.07) ^a^ |
|  | BARxCROP | 0.88 (± 0.27) ^a^ | 0.74 (± 0.10) ^a^ | 0.17 (± 0.17) ^a^ | 1.00 (± 0.15) ^a^ |
|  | RCUxCROP | 0.90 (± 0.27) ^a^ | 0.89 (± 0.09) ^a^ | 0.24 (± 0.17) ^a^ | 0.49 (± 0.15) ^b^ |
|  | CROPxBAR | 1.19 (± 0.27) ^a^ | 0.89 (± 0.08) ^a^ | 0.77 (± 0.13) ^a^ | 1.00 (± 0.07) ^a^ |
|  | CROPxRCU | 0.86 (± 0.27) ^a^ | 0.78 (± 0.08) ^a^ | 0.37 (± 0.13) ^a^ | 1.00 (± 0.09) ^a^ |
|  | VOL | 0.84 (± 0.30) ^a^ | 0.07 (± 0.09) ^b^ | - | - |
|  | BRW | 0.69 (± 0.27) ^a^ | 0.75 (± 0.08) ^a^ | 0.39 (± 0.13) ^a^ | 0.89 (± 0.07) ^a^ |

Table S3. Fitness-related traits in the first year (2019-2020) of wild, weedy, cultivated and crop-wild hybrid populations throughout the life cycle stages under agrestal (AGR1 and AGR2) or ruderal (RUD) environments. Variables are expressed as number of plants (except *flowering,* expressed as degree-day from first flowering plant) ± standard error. Different letters mean significant differences by Tukey’s comparison test.

| Environment | Population | Fall emergence (6 weeks) | Spring emergence | Established plants | Reproductive plants | Flowering (DD) | Heads per plot | Heads per plant |
| --- | --- | --- | --- | --- | --- | --- | --- | --- |
| AGR1 | BAR | 122.80 (± 12.06) ^b^ | 28.20 (± 3.89) ^b^ | 7.80 (± 2.89) ^ac^ | 8.00 (± 2.92) ^ac^ | 578.07 (± 83.77) ^a^ | 516.20 (± 97.98) ^a^ | 64.52 (± 10.08) ^c^ |
|  | RCU | 67.40 (± 2.25) ^c^ | 20.40 (± 3.82) ^bc^ | 5.40 (± 3.44) ^bd^ | 5.40 (± 3.44) ^bd^ | 1124.08 (± 210.23) ^b^ | 382.20 (± 81.44) ^a^ | 37.58 (± 7.58) ^bc^ |
|  | BAR x CROP | 167.80 (± 8.87) ^a^ | 11.40 (± 3.43) ^ce^ | 4.60 (± 1.66) ^cd^ | 4.60 (± 1.57) ^cd^ | 573.19 (± 15.37) ^a^ | 378.20 (± 118.14) ^a^ | 70.9 (± 17.5) ^c^ |
|  | RCU x CROP | 165.60 (± 26.72) ^a^ | 5.00 (± 1.22) ^e^ | 1.00 (± 0.32) ^d^ | 1.00 (± 0.32) ^de^ | 795.16 (± 68.35) ^ab^ | 96.80 (± 40.61) ^b^ | 96.8 (± 20.06) ^c^ |
|  | CROP x BAR | 30.00 (± 8.46) ^c^ | 15.20 (± 3.89) ^cd^ | 10.20 (± 2.60) ^ac^ | 9.80 (± 2.24) ^ac^ | 475.66 (± 79.48) ^a^ | 314.80 (± 72.10) ^a^ | 25.91 (± 3.21) ^bc^ |
|  | CROP x RCU | 20.00 (± 4.11) ^e^ | 31.20 (± 8.18) ^b^ | 10.60 (± 1.75) ^ab^ | 10.60 (± 1.75) ^ab^ | 526.77 (± 111.17) ^a^ | 376.40 (± 75.27) ^a^ | 30.56 (± 3.8) ^bc^ |
|  | VOL | 38.20 (± 11.78) ^c^ | 11.00 (± 4.60) ^de^ | 1.40 (± 0.75) ^d^ | 1.00 (± 0.77) ^e^ | 423.30 (± 319.00) ^a^ | 1.40 (± 0.87) ^c^ | 1.4 (± 0.4) ^a^ |
|  | BRW | 38.40 (± 6.01) ^d^ | 102.40 (± 9.94) ^a^ | 15.00 (± 5.00) ^a^ | 14.80 (± 5.05) ^a^ | 808.20 (± 116.24) ^ab^ | 290.40 (± 46.69) ^a^ | 15.05 (± 1.75) ^b^ |
| AGR2 | BAR | 117.00 (± 9.79) ^b^ | 19.80 (± 2.42) ^cd^ | 12.40 (± 1.33) ^bc^ | 8.60 (± 1.53) ^bd^ | 1086.22 (± 78.22) ^de^ | 256.80 (± 54.64) ^ab^ | 27.72 (± 3.7) ^d^ |
|  | RCU | 61.40 (± 6.65) ^c^ | 40.60 (± 6.86) ^b^ | 22.40 (± 6.52) ^b^ | 16.00 (± 4.53) ^b^ | 1364.05 (± 0.00) ^e^ | 332.60 (± 63.41) ^a^ | 12.47 (± 1.25) ^c^ |
|  | BAR x CROP | 177.20 (± 19.49) ^a^ | 15.60 (± 3.40) ^cd^ | 4.20 (± 1.16) ^d^ | 4.60 (± 1.29) ^d^ | 630.20 (± 87.16) ^bc^ | 145.80 (± 46.47) ^b^ | 27.81 (± 5.22) ^cd^ |
|  | RCU x CROP | 198.00 (± 12.29) ^ab^ | 12.60 (± 3.23) ^cd^ | 5.60 (± 1.12) ^cd^ | 5.20 (± 0.97) ^cd^ | 557.35 (± 50.72) ^abc^ | 165.00 (± 48.96) ^b^ | 31.73 (± 5.8) ^cd^ |
|  | CROP x BAR | 45.40 (± 7.22) ^d^ | 24.00 (± 3.30) ^bc^ | 19.60 (± 3.07) ^b^ | 18.20 (± 3.12) ^ab^ | 410.80 (± 0.00) ^ab^ | 371.60 (± 53.99) ^a^ | 20.41 (± 1.92) ^cd^ |
|  | CROP x RCU | 17.60 (± 4.17) ^d^ | 23.20 (± 8.24) ^cd^ | 17.40 (± 6.20) ^b^ | 14.80 (± 5.12) ^bc^ | 349.67 (± 61.13) ^a^ | 379.40 (± 72.95) ^a^ | 21.21 (± 2.45) ^cd^ |
|  | VOL | 46.40 (± 17.91) ^d^ | 10.40 (± 4.26) ^d^ | 6.60 (± 3.09) ^cd^ | 7.00 (± 3.46) ^cd^ | 328.51 (± 111.17) ^a^ | 15.20 (± 5.09) ^c^ | 1.75 (± 0.23) ^a^ |
|  | BRW | 43.80 (± 6.48) ^d^ | 129.40 (± 17.01) ^a^ | 52.50 (± 5.60) ^a^ | 29.40 (± 4.12) ^a^ | 808.20 (± 0.00) ^cd^ | 273.20 (± 49.88) ^ab^ | 6.32 (± 0.48) ^b^ |
| RUD | BAR | 115.00 (± 9.88) ^a^ | 8.40 (± 5.17) ^cd^ | 12.60 (± 5.10) ^cd^ | 12.40 (± 4.82) ^c^ | 1224.78 (± 86.27) ^d^ | 216.60 (± 55.06) ^ab^ | 14.32 (± 1.61) ^c^ |
|  | RCU | 49.80 (± 3.93) ^b^ | 27.00 (± 6.57) ^b^ | 32.60 (± 11.61) ^ab^ | 27.20 (± 10.10) ^ab^ | 1399.26 (± 70.09) ^d^ | 279.80 (± 26.05) ^a^ | 7.7 (± 0.63) ^b^ |
|  | BAR x CROP | 120.20 (± 11.91) ^a^ | 4.60 (± 1.44) ^d^ | 5.60 (± 3.14) ^d^ | 3.60 (± 1.69) ^d^ | 736.87 (± 47.43) ^ac^ | 29.40 (± 14.29) ^c^ | 7 (± 0.85) ^bc^ |
|  | RCU x CROP | 151.20 (± 22.95) ^a^ | 4.20 (± 0.97) ^d^ | 3.80 (± 1.39) ^d^ | 3.20 (± 1.07) ^d^ | 762.75 (± 41.08) ^bc^ | 25.60 (± 7.05) ^c^ | 8 (± 1.73) ^bc^ |
|  | CROP x BAR | 64.00 (± 18.46) ^b^ | 17.40 (± 6.33) ^bc^ | 17.60 (± 5.25) ^bc^ | 15.20 (± 4.33) ^bc^ | 615.43 (± 65.73) ^ab^ | 164.60 (± 38.97) ^b^ | 9.67 (± 0.72) ^c^ |
|  | CROP x RCU | 35.80 (± 19.72) ^b^ | 17.60 (± 1.96) ^b^ | 16.20 (± 4.18) ^bc^ | 12.20 (± 2.56) ^bc^ | 660.44 (± 169.59) ^ab^ | 154.80 (± 28.16) ^b^ | 10.7 (± 0.81) ^c^ |
|  | VOL | 48.60 (± 24.72) ^b^ | 7.60 (± 6.36) ^d^ | 6.60 (± 5.61) ^d^ | 1.60 (± 1.60) ^d^ | 457.50 (± 58.36) ^a^ | 1.60 (± 1.60) ^d^ | 1 (± 0) ^a^ |
|  | BRW | 36.40 (± 8.72) ^b^ | 70.40 (± 3.01) ^a^ | 49.60 (± 11.68) ^a^ | 35.00 (± 6.88) ^a^ | 925.81 (± 190.22) ^c^ | 212.60 (± 37.29) ^ab^ | 4.81 (± 0.22) ^b^ |

Table S4. Fitness-related traits in the second year (2020-2021) of the wild, weedy, cultivated and crop-wild hybrid populations throughout the life cycle stages under agrestal (AGR1 and AGR2) or ruderal (RUD) environments. Variables are expressed as the mean number of plants (except *flowering,* expressed as sum of degree-day) ± standard error. Different letters mean significant differences by Tukey’s comparison test.

| Environment | Population | Fall emergence | Spring emergence | Established plants | Reproductive plants | Flowering | Heads per plot | Heads per plant |
| --- | --- | --- | --- | --- | --- | --- | --- | --- |
| AGR1 | BAR | 6.80 (± 1.52) ^b^ | 10.40 (± 2.76) ^bc^ | 10.00 (± 2.30) ^bc^ | 10.00 (± 2.30) ^bc^ | 369,07 (± 50.15) ^a^ | 373.00 (± 29.75) ^bc^ | 28.46 (± 3.72) ^bc^ |
|  | RCU | 17.00 (± 2.91) ^a^ | 26.60 (± 3.08) ^a^ | 26.60 (± 3.07) ^a^ | 25.20 (± 3.25) ^a^ | 836,44 (± 30.31) ^a^ | 736.20 (± 48.37) ^a^ | 23.63 (± 2.06) ^b^ |
|  | BARxCROP | 2.20 (± 0.73) ^c^ | 5.20 (± 2.75) ^cd^ | 5.40 (± 3.20) ^cd^ | 5.40 (± 3.20) ^cd^ | 370,64 (± 75.93) ^a^ | 265.60 (± 116.43) ^bc^ | 34.91 (± 5.68) ^bd^ |
|  | RCUxCROP | 12.00 (± 5.05) ^ab^ | 17.67 (± 6.70) ^ab^ | 16.00 (± 6.38) ^ab^ | 15.67 (± 5.95) ^ab^ | 355,13 (± 113.97) ^a^ | 418.67 (± 134.66) ^b^ | 25.06 (± 2.52) ^bc^ |
|  | CROPxBAR | 2.60 (± 1.12) ^c^ | 3.60 (± 1.88) ^d^ | 2.80 (± 1.11) ^d^ | 2.80 (± 1.11) ^d^ | 628,82 (± 276.98) ^a^ | 209.00 (± 41.26) ^c^ | 74.64 (± 16.48) ^d^ |
|  | CROPxRCU | 2.20 (± 0.58) ^c^ | 5.60 (± 1.36) ^cd^ | 4.80 (± 1.02) ^cd^ | 4.80 (± 1.02) ^cd^ | 831,47 (± 283.78) ^a^ | 220.00 (± 57.17) ^c^ | 45.83 (± 7.03) ^cd^ |
|  | VOL | 1.00 (± 0.57) ^c^ | 0.00 (± 0.00) ^e^ | 0.00 (± 0.00) | - | - | - | - |
|  | BRW | 6.60 (± 0.81) ^b^ | 18.80 (± 2.94) ^a^ | 17.40 (± 2.25) ^ab^ | 16.20 (± 1.90) ^ab^ | 472,57 (± 98.72) ^a^ | 274.40 (± 39.51) ^bc^ | 10.75 (± 1.17) ^a^ |
| AGR2 | BAR | 5.00 (± 1.30) ^ab^ | 6.20 (± 2.17) ^bc^ | 4.60 (± 1.12) ^bc^ | 4.60 (± 1.12) ^bc^ | 117,67 (± 44.07) ^a^ | 455.80 (± 99.75) ^ac^ | 99.08 (± 10.94) ^b^ |
|  | RCU | 12.80 (± 5.19) ^a^ | 15.00 (± 4.86) ^ab^ | 12.40 (± 4.47) ^ab^ | 11.40 (± 4.02) ^ab^ | 346,73 (± 51.77) ^b^ | 722.60 (± 141.32) ^a^ | 63.38 (± 7.34) ^ab^ |
|  | BARxCROP | 0.40 (± 0.40) ^c^ | 1.20 (± 0.80) ^d^ | 0.80 (± 0.58) ^d^ | 1.00 (± 0.60) ^d^ | 139,65 (± 0) ^ab^ | 140.4 (± 92.87) ^d^ | 140.4 (± 30.17) ^b^ |
|  | RCUxCROP | 2.40 (± 1.02) ^bc^ | 2.20 (± 0.86) ^cd^ | 2.20 (± 0.86) ^cd^ | 2.00 (± 0.95) ^cd^ | 110,8 (± 28.85) ^a^ | 243.6 (± 101.47) ^bd^ | 121.8 (± 19.26) ^b^ |
|  | CROPxBAR | 1.80 (± 0.58) ^bc^ | 1.60 (± 0.51) ^cd^ | 1.40 (± 0.51) ^cd^ | 1.40 (± 0.51) ^cd^ | 112,175 (± 56.44) ^ab^ | 162.2 (± 64.59) ^cd^ | 115.85 (± 38.36) ^ab^ |
|  | CROPxRCU | 2.00 (± 1.26) ^bc^ | 4.40 (± 2.50) ^cd^ | 3.20 (± 1.82) ^cd^ | 3.00 (± 1.67) ^cd^ | 268,85 (± 129.2) ^ab^ | 276.8 (± 124.59) ^bd^ | 92.26 (± 22.81) ^ab^ |
|  | VOL | 2.00 (± 0.54) ^bc^ | 2.40 (± 0.87) ^cd^ | 0.00 (± 0.00) | - | - | - | - |
|  | BRW | 4.00 (± 0.70) ^b^ | 18.20 (± 4.57) ^a^ | 14.20 (± 3.50) ^a^ | 12.20 (± 2.35) ^a^ | 291,55 (± 48.92) ^a^ | 519.20 (± 97.82) ^ab^ | 39.16 (± 4.98) ^a^ |
| RUD | BAR | 21.00 (± 4.52) ^a^ | 24.40 (± 3.93) ^a^ | 14.00 (± 4.77) ^a^ | 13.20 (± 4.53) ^a^ | 1101,84 (± 24.81) ^de^ | 246.60 (± 51.81) ^a^ | 15.56 (± 1.83) ^a^ |
|  | RCU | 11.80 (± 3.16) ^b^ | 9.80 (± 2.31) ^b^ | 6.60 (± 2.25) ^ab^ | 6.00 (± 1.87) ^ab^ | 1211,88 (± 52.32) ^e^ | 162.60 (± 26.58) ^a^ | 20.82 (± 4.19) ^a^ |
|  | BARxCROP | 4.00 (± 1.76) ^cd^ | 5.33 (± 2.69) ^bc^ | 1.67 (± 1.29) ^c^ | 1.67 (± 1.29) ^c^ | 260,55 ^ab^ | 32.67 (± 25.30) ^bc^ | 19.6 (± 5.53) ^a^ |
|  | RCUxCROP | 2.70 (± 0.87) ^d^ | 3.67 (± 1.44) ^c^ | 0.67 (± 0.51) ^c^ | 0.33 (± 0.26) ^c^ | 24,25 ^a^ | 5.00 (± 3.87) ^c^ | 1.17 ^a^ |
|  | CROPxBAR | 1.40 (± 0.50) ^d^ | 2.20 (± 0.37) ^c^ | 1.60 (± 0.40) ^bc^ | 1.60 (± 0.40) ^bc^ | 621,28 (± 70.58) ^bc^ | 71.20 (± 25.22) ^b^ | 44.5 (± 16.62) ^a^ |
|  | CROPxRCU | 3.00 (± 1.37) ^d^ | 3.20 (± 1.28) ^c^ | 1.20 (± 0.73) ^c^ | 1.20 (± 0.73) ^c^ | 896,5 (± 162.18) ^cd^ | 28.2 (± 17.49) ^bc^ | 23.5 (± 9.36) ^a^ |
|  | VOL | 0.20 (± 0.20) ^e^ | 0.40 (± 0.24) ^d^ | 0.00 (± 0.00) | - | - | - | - |
|  | BRW | 9.80 (± 1.49) ^bc^ | 25.60 (± 4.33) ^a^ | 9.20 (± 2.48) ^a^ | 7.80 (± 1.82) ^a^ | 984,34 (± 54.81) ^de^ | 183.80 (± 48.44) ^a^ | 21.63 (± 3.01) ^a^ |
